# Neutrophil‐to‐lymphocyte ratio and its changes predict the 3‐month outcome and mortality in acute ischemic stroke patients after intravenous thrombolysis

**DOI:** 10.1002/brb3.3162

**Published:** 2023-07-20

**Authors:** Qiong Wu, Hui‐Sheng Chen

**Affiliations:** ^1^ Graduate College Liaoning University of Traditional Chinese Medicine Shenyang China; ^2^ Department of Neurology General Hospital of Northern Theater Command Shenyang China

**Keywords:** 3‐month outcome, acute ischemic stroke, intravenous thrombolysis, mortality, neutrophil‐to‐lymphocyte ratio

## Abstract

**Background and purpose:**

The neutrophil‐to‐lymphocyte ratio (NLR) has been demonstrated as a prognostic inflammatory biomarker in ischemic stroke. The study aimed to investigate the association of NLR and its dynamic change with long‐term outcome and mortality in acute ischemic stroke (AIS) patients who received intravenous thrombolysis (IVT).

**Methods:**

From a prospective cohort, AIS patients receiving IVT (alteplase, 0.9 mg/kg) with complete NLR data were retrospectively screened. Based on 3‐month modified Rankin scale score (mRS), patients were classified into good group (mRS 0–1) and poor outcome group (mRS 2–6), or survival group (mRS 0–5) and death group (mRS 6). Multivariate logistic regression analysis and receiver operating curve were used to identify prognostic factors and their predictive powers.

**Results:**

A total of 259 eligible patients were enrolled in our study. Logistic regression analysis showed that NLR at 24 h (adjusted odds ratio [aOR] 1.182), 12 days (aOR 1.218) after IVT was independent predictors of 3‐month outcome with the AUC of 0.815, 0.820, respectively, whereas NLR at 24 h (aOR 1.17), 12 days (aOR 1.252) after IVT and percentage changes of NLR between admission and 24 h after IVT (aOR 1.214), and between admission and 12 days after IVT (aOR 1.233) were independent predictors of 3‐month mortality with the AUCs of 0.86, 0.902, 0.814, and 0.855, respectively.

**Conclusion:**

The comprehensive report suggests that NLR and its dynamic changes are associated with 3‐month outcome and mortality in AIS patients after IVT with good predictive powers.

## Graphical abstract

The study aimed to investigate the association of NLR and its dynamic change with long‐term outcome and mortality in acute ischemic stroke (AIS) patients who received intravenous thrombolysis (IVT). The results suggest that NLR and its dynamic changes are associated with 3‐month outcome and mortality in AIS patients after IVT with good predictive powers.

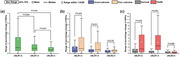

## 1 INTRODUCTION

Based on data from the Global Burden of Disease Study 2019 (GBD 2019 Stroke Collaborators, 2021), stroke is the second leading cause of disability and mortality worldwide, and the prevalence of stroke in China has continued to increase during 2013−2019 (Tu et al., 2022). As the most common type of stroke, acute ischemic stroke (AIS) possesses high morbidity and mortality (GBD 2019 Stroke Collaborators, 2021; Tu et al., 2022). Intravenous thrombolysis (IVT) is one of effective treatments for AIS, but about 1/3 patients get good prognosis (Kastrup et al., 2017). It has been a hot topic about how to accurately predict the outcome of AIS patients after IVT. A lot of prognostic factors have been identified, for example, advanced age (Emberson et al., 2014), higher National Institutes of Health Stroke Scale (NIHSS) score (Kawiorski et al., 2016), and neuroinflammatory response (Kim et al., 2016; Parikh et al., 2020; Ramiro et al., 2018) are associated with poor outcome after thrombolysis in stroke patients. Due to its low cost and easy availability in clinical practice, neutrophil‐to‐lymphocyte ratio (NLR) has been reported to have strong prognostic effects on clinical outcomes of AIS patients without reperfusion treatment, for example, the association of NLR with short‐term mortality, stroke severity on admission, unfavorable functional outcome, and recurrent ischemic stroke (Goyal et al., 2018; Sharma et al., 2022; Tokgoz et al., 2013; Wang et al., 2019). In AIS patients treated with IVT, most of these studies focused on the predictive effects of NLR at admission and within 24 h after IVT (Brooks et al., 2014; Li et al., 2022; Maestrini et al., 2015), and only a few studies expanded the time points to 48 h, 3 days, or even 7 days (Guo et al., 2016; Weng et al., 2021); however, there is a lack of studies to systemically investigate the prognostic roles of NLR at extended time point and their dynamic changes in AIS patients after IVT.

In this study, we aimed to investigate the association of NLR at admission, 24 h and 12 days after IVT and their dynamic changes with 3‐month outcome and mortality in AIS patients after IVT.

## 2 MATERIALS AND METHODS

### 2.1 Subjects

Based on the INTRECIS database (Wang et al., 2021), we retrospectively screened consecutive AIS patients receiving IVT from October 2016 and September 2019. The inclusion criteria were (1) age ≥18 years; (2) diagnosis of acute AIS; (3) treatment with the standard dose of rt‐PA (alteplase, 0.9 mg/kg up to a maximum of 90 mg/kg) within 4.5 h of symptom onset; (4) the data of neutrophil and lymphocyte on admission, 24 h and 12 days after IVT were available; (5) complete clinical data. Patients with any of the following conditions were excluded: (1) receiving endovascular therapy; (2) combined acute or chronic infection; (3) combined severe heart, lung, liver, and kidney diseases; (4) lack of complete clinical data. The INTRECIS study was centrally approved by the General Hospital of the Northern Theater Command (IRB: K201707) and is registered at Clinicaltrials.gov (unique identifier: NCT02854592), and all patients provided written informed consent.

### 2.2 Data collection and definition

From EDC system, we collected data, including demographics (age, gender, height, and weight), risk factors (hypertension, diabetes mellitus, coronary heart disease, atrial fibrillation, previous stroke, alcohol consumption, and smoking), clinical data on admission (NIHSS score, heart rate, systolic blood pressure, diastolic blood pressure, and random blood glucose), etiological classification, and modified Rankin Scale (mRS) score at 90 days. In particular, blood samples of all patients were collected and analyzed at three time points: time1 was on admission, time2 was 24 h after IVT, and time3 was 12 days after IVT.

Hypertension, diabetes mellitus, and coronary heart disease or atrial fibrillation were determined if a definite history existed or if the condition was definitely diagnosed at discharge. Smoker is definite as smoking until the symptom onset of stroke or quit smoking within 1 year, and drinker is definite as alcohol abuse >2 U/day. The NLR was calculated by the neutrophil count/lymphocyte count formula: cNLR1–2 was calculated by (NLR at time2 − NLR at time1)/NLR at time1 × 100%, cNLR1–3 by (NLR at time3 − NLR at time1)/NLR at time1 × 100%, and cNLR2–3 by (NLR at time3 − NLR at time2)/NLR at time2 × 100%.

### 2.3 Outcome assessments

All patients were assessed at 3‐month using mRS. Good outcome was defined as a functional independence (a mRS score of 0 or 1), whereas a score of 2 or greater on the mRS was defined as poor outcome. Survival was defined as a score of 1–5 on the mRS, whereas a score of 6 on the mRS was defined as death group.

### 2.4 Statistical analysis

All data analyses were performed by SPSS 26.0. Distribution normality was tested by the Kolmogorov–Smirnov test. Continuous variables were exhibited by mean ± standard deviation or medians and interquartile range (IQR), which were analyzed by independent test or Mann–Whitney *U*‐test, respectively. Categorical data were represented as frequencies (percentages) and were analyzed using the chi‐square test or Fisher's exact test. Relations between NLR at different time points were analyzed using the generalized estimating equation. The relationship between the percentage change between the different time points of NLR were analyzed by paired *t* test. Univariate and multivariate logistic regression analyses were used to calculate the relationship between NLR and poor outcome or death. For multivariate analysis, the pool of covariates was determined based on univariate analysis at *p* < .1 level and previous literature. The results were reported as an odds ratio (OR) and 95% confidence interval (CI). Two‐sided values of *p* < .05 were considered statistically significant. The receiver operating curve (ROC) was used to demonstrate the predictive power of NLR.

## 3 RESULTS

### 3.1 Baseline characteristics of the patients

**FIGURE 1:**
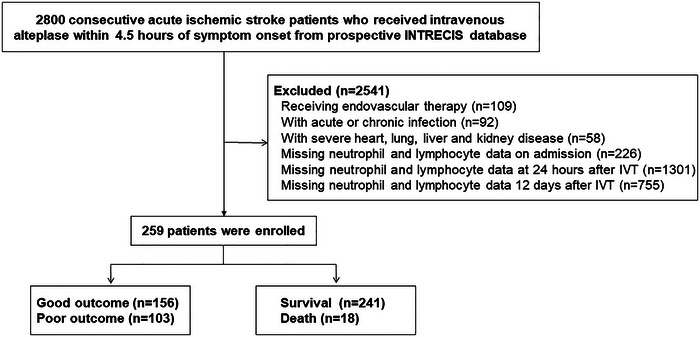
Flow diagram showing the patient selection process. IVT, intravenous thrombolysis.

From 2800 AIS patients receiving intravenous rt‐PA within 4.5 h of symptom onset, 2541 patients meeting the exclusion criteria were excluded. Finally, 259 eligible patients were enrolled in our study (Figure 1). The baseline characteristics and outcomes of the study population are detailed in Table 1. There were 175 males (67.6%); the median admission NIHSS was 7 (IQR 4–14) and 108 (69.2%) with good outcome. Compared with good outcome group, patients with poor outcome had higher NIHSS score and lower weight and body mass index, more prevalence of atrial fibrillation (all *p* < .05, Table 1). Neutrophil and lymphocyte at 24 h and 12 days after IVT, NLR2, NLR3, cNLR1–2, and cNLR1–3 were higher in poor versus good outcome group (all *p* < .001, Table 2). Compared with survival group, patients with death group had higher NIHSS score and heart rate, more prevalence of atrial fibrillation (all *p* < .05, Table 1). Neutrophil and lymphocyte at 24 h and 12 days after IVT, NLR2, NLR3, cNLR1–2, and cNLR1–3 were higher in survival versus death group (all *p* < .05, Table 2).

**TABLE 1:** Comparison of clinical characteristics between good and poor outcomes (or survival and death).

					Survival	Death	
Variable	Total (*n* = 259)	Good outcome mRS (0–1) (*n* = 156)	Poor outcome mRS (2–6) (*n* = 103)	*p*	(*n* = 241)	(*n* = 18)	*p*
**Demographics, mean ± SD/*N*.(%)**							
Gender (F/M)	175 (67.6)/84 (32.4)	108 (69.2)/48 (30.8)	67 (65)/36 (35)	.482^c^	164 (68)/77 (32)	11 (61.1)/7 (38.9)	.544^c^
Age (years)	66.27 ± 11.95	65.62 ± 12.15	67.26 ± 11.64	.281^a^	65.88 ± 11.92	71.56 ± 11.5	.052^a^
Height (cm)	167.63 ± 7.36	168.07 ± 6.97	166.97 ± 7.90	.243^a^	167.76 ± 7.29	165.96 ± 8.26	.317^a^
Weight (kg)	67.18 ± 12.63	69.07 ± 12.57	64.33 ± 12.24	**.003** ^a^	67.4 ± 12.68	64.29 ± 12.01	.314^a^
BMI (kg/m^2^)	23.8 ± 3.64	24.36 ± 3.72	22.96 ± 3.35	**.002** ^a^	23.85 ± 3.68	23.19 ± 3.04	.464^a^
**Underlying diseases, *N*.(%)**							
Hypertension	154 (59.5)	95 (60.9)	59 (57.3)	.562^c^	142 (58.9)	12 (66.7)	.519^c^
Diabetes mellitus	58 (22.4)	38 (24.4)	20 (19.4)	.350^c^	56 (23.2)	2 (11.1)	.234^c^
Coronary heart disease	33 (12.7)	18 (11.5)	15 (14.6)	.475^c^	29 (12)	4 (22.2)	.211^c^
Atrial fibrillation	40 (15.4)	18 (11.5)	22 (21.4)	**.032** ^c^	33 (13.7)	7 (38.9)	**.004** ^c^
Previous stroke	60 (23.2)	36 (23.1)	24 (23.3)	.967^c^	53 (22)	7 (38.9)	.101^c^
Current smoking	106 (40.9)	63 (40.4)	43 (41.7)	.827^c^	101 (41.9)	5 (27.8)	.240^c^
Current drinking	63 (24.3)	38 (24.4)	25 (24.3)	.987^c^	61 (25.3)	2 (11.1)	.176^c^
**Clinical assessment, mean ± SD/median (IQR)**							
Admission NIHSS score	7 (4, 14)	5 (3, 8)	12 (8, 19)	**<.001** ^b^	9.25 ± 8.34	17.78 ± 10.44	**<.001** ^a^
TOAST subtype				**.001** ^c^			.061^c^
LAA	121 (46.7)	69 (44.2)	52 (50.5)		111 (46.1)	10 (55.6)	
CE	54 (20.8)	25 (16)	29 (28.2)		47 (19.5)	7 (38.9)	
SAO	71 (27.4)	57 (36.5)	14 (13.6)		71 (29.5)	0 (0)	
SOE	3 (1.2)	1 (0.6)	2 (1.9)		3 (1.2)	0 (0)	
SUE	10 (3.9)	4 (2.6)	6 (5.8)		9 (3.7)	1 (5.6)	
Heart rate (/min)	80.2 ± 16.03	78.66 ± 16.38	82.54 ± 15.26	.056^a^	79.56 ± 15.74	88.89 ± 17.72	**.017** ^a^
SBP (mmHg)	154 (140, 168)	150 (140, 166)	156 (140, 170)	.259^b^	153.95 ± 22.73	157.5 ± 25.65	.528^a^
DBP (mmHg)	88.29 ± 14.15	88.20 ± 14.25	88.42 ± 14.07	.903^a^	88.41 ± 14.09	86.61 ± 15.29	.604^a^
Random blood glucose (mmol/L)	8.48 ± 4.62	8.37 ± 5.23	8.65 ± 3.49	.655^a^	8.49 ± 4.75	8.36 ± 2.78	.913^a^

Abbreviations: CE, cardioembolism; DBP, diastolic blood pressure; IQR, interquartile range; LAA, large artery atherosclerosis; mRS, modified Rankin scale score; NIHSS, National Institute of Health Stroke Scale; SAO, small‐artery occlusion; SBP, systolic blood pressure; SD, standard deviation; SOE, stroke of other determined etiology; SUE, stroke of undetermined etiology; TOAST, Trial of Org 10 172 in acute stroke treatment.

^a^ *t* Test.

^b^ Mann–Whitney *U*‐test.

^c^ *x* ^2^ test.

**TABLE 2:** Comparison of inflammatory biomarkers between good and poor outcomes (or survival and death).

					Survival	Death	
Variable	Total (*n* = 259)	Good outcome mRS (0–1) (*n* = 156)	Poor outcome mRS (2–6) (*n* = 103)	*p*	(*n* = 241)	(*n* = 18)	*p*
**Neutrophil (10^9^/L)**							
Neutrophil (Admission)	5.76 ± 2.88	5.53 ± 2.82	6.09 ± 2.94	.126^a^	5.74 ± 2.89	6 ± 2.74	.710^a^
Neutrophil (24 h after IVT)	6.11 (4.5, 8.75)	5.51 (4, 7.37)	8.1 (5.81, 10.33)	**<.001** ^b^	6.68 ± 3.11	9.67 ± 2.78	**<.001** ^a^
Neutrophil (12 ± 2 days after IVT)	5.3 (3.7, 6.82)	4.52 (3.52, 5.75)	6.3 (4.5, 9.1)	**<.001** ^b^	5.1 (3.6, 6.45)	10.96 (7.27, 14.21)	**<.001** ^b^
**Lymphocyte (10^9^/L)**							
Lymphocyte1 (admission)	2.12 ± 1.42	2.16 ± 1.40	2.06 ± 1.44	.551^a^	2.11 ± 1.36	2.31 ± 2.09	.545^a^
Lymphocyte2 (24 h after IVT)	1.49 (1.1, 1.99)	164 (1.18, 2.12)	1.27 (0.9, 1.7)	**<.001** ^b^	1.7 ± 1.05	1.14 ± 0.62	**.029** ^a^
Lymphocyte3 (12 ± 2 days after IVT)	1.78 ± 1.01	1.96 ± 1.16	1.51 ± 0.66	**<.001** ^b^	1.84 ± 1.03	1.06 ± 0.4	**.002** ^a^
**NLR (neutrophil‐to‐lymphocyte ratio), mean** ± **SD/median (IQR)**							
NLR1 (admission)	3.94 ± 4.09	3.63 ± 4.06	4.40 ± 4.12	.137^a^	3.88 ± 3.98	4.73 ± 5.49	.398^a^
NLR2 (24 h after IVT)	4.41 (2.45, 6.86)	3.4 (2.1, 5.45)	6.31 (3.66, 9,1)	**<.001** ^b^	4.23 (2.37, 6.67)	9.29 (4.81, 19.44)	**<.001** ^b^
NLR3 (12 ± 2 days after IVT)	3.13 (1.94, 4.85)	2.66 (1.71, 3.92)	4.52 (2.71, 7.48)	**<.001** ^b^	2.96 (1.88, 4.62)	11.45 (5.22, 19.39)	**<.001** ^b^
cNLR1–2 (×100%)	0.58 (0.27, 1.6)	0.46 (0.22, 1.18)	0.83 (0.48, 2.76)	**<.001** ^b^	0.56 (0.25, 1.47)	1.45 (0.45, 6.07)	**.007** ^b^
cNLR1–3 (×100%)	0.51 (0.28, 0.88)	0.43 (0.25, 0.69)	0.67 (0.34, 1.88)	**<.001** ^b^	0.48 (0.27, 0.78)	2.49 (1.06, 5.92)	**<.001** ^b^
cNLR2–3 (×100%)	0.49 ± 0.78	0.47 ± 0.95	0.51 ± 0.41	.705^a^	0.48 ± 0.8	0.51 ± 0.51	.888^a^

Abbreviations: cNLR1–2, percentage change between NLR1 and NLR2; cNLR1–3, percentage change between NLR1 and NLR3; cNLR2–3, percentage change between NLR2 and NLR3; IQR, interquartile range; IVT, intravenous thrombolysis; mRS, modified Rankin scale score; SD, standard deviation.

^a^ T test.

^b^ Mann–Whitney *U*‐test.

### 3.2 The association of NLR and its dynamic change with poor outcome or death

**FIGURE 2:**
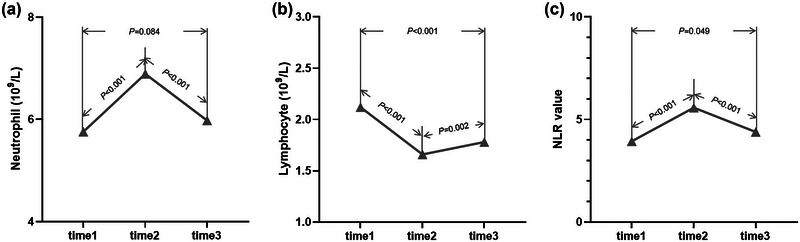
Inflammatory biomarkers at different time points.(a) Showing the dynamic change of neutrophil. (b) Showing the dynamic change of lymphocyte. (c) Showing the dynamic change of NLR. NLR, neutrophil‐to‐lymphocyte ratio; time1, admission; time2, 24 h after intravenous thrombolysis; time3, 12 ± 2 days after intravenous thrombolysis.

**FIGURE 3:**
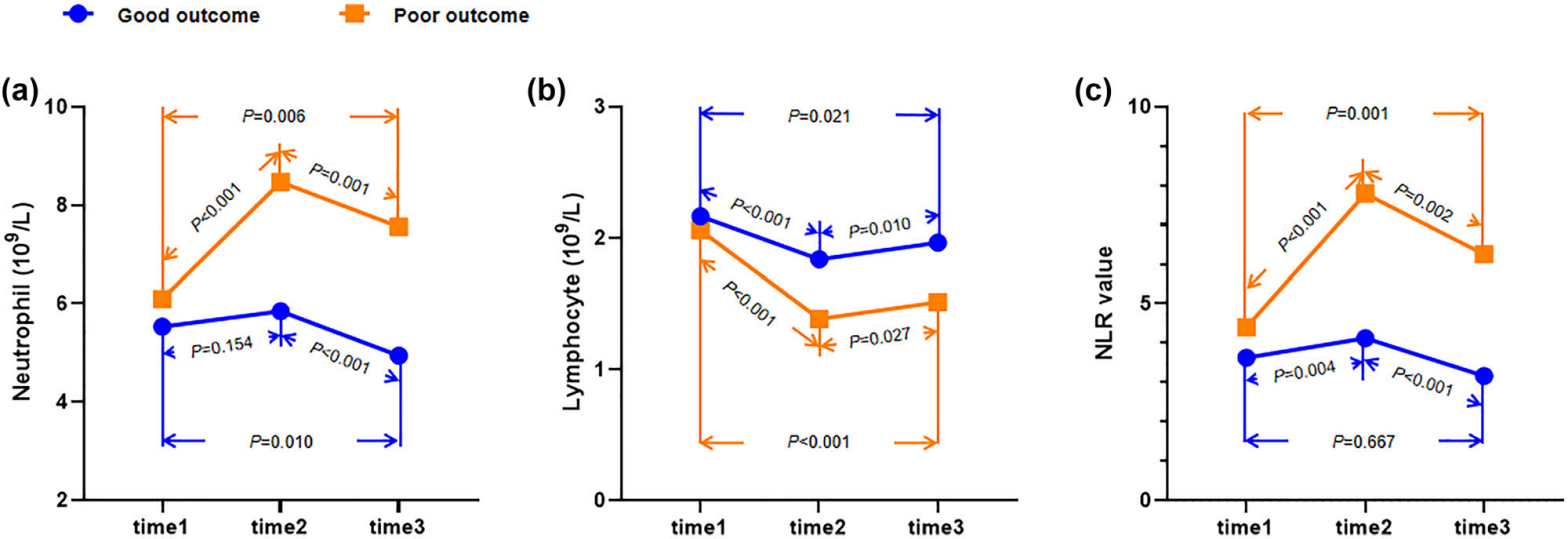
Inflammatory biomarkers at different time points between good and poor outcome groups. (a) Showing the dynamic changes of Neutrophil levels between good and poor outcome groups. (b) Showing the dynamic changes of lymphocyte levels between good and poor outcome groups. (c) Showing the dynamic changes of NLR levels between good and poor outcome groups. NLR, neutrophil‐to‐lymphocyte ratio; time1, admission; time2, 24 h after intravenous thrombolysis; time3, 12 ± 2 days after intravenous thrombolysis.

**FIGURE 4:**
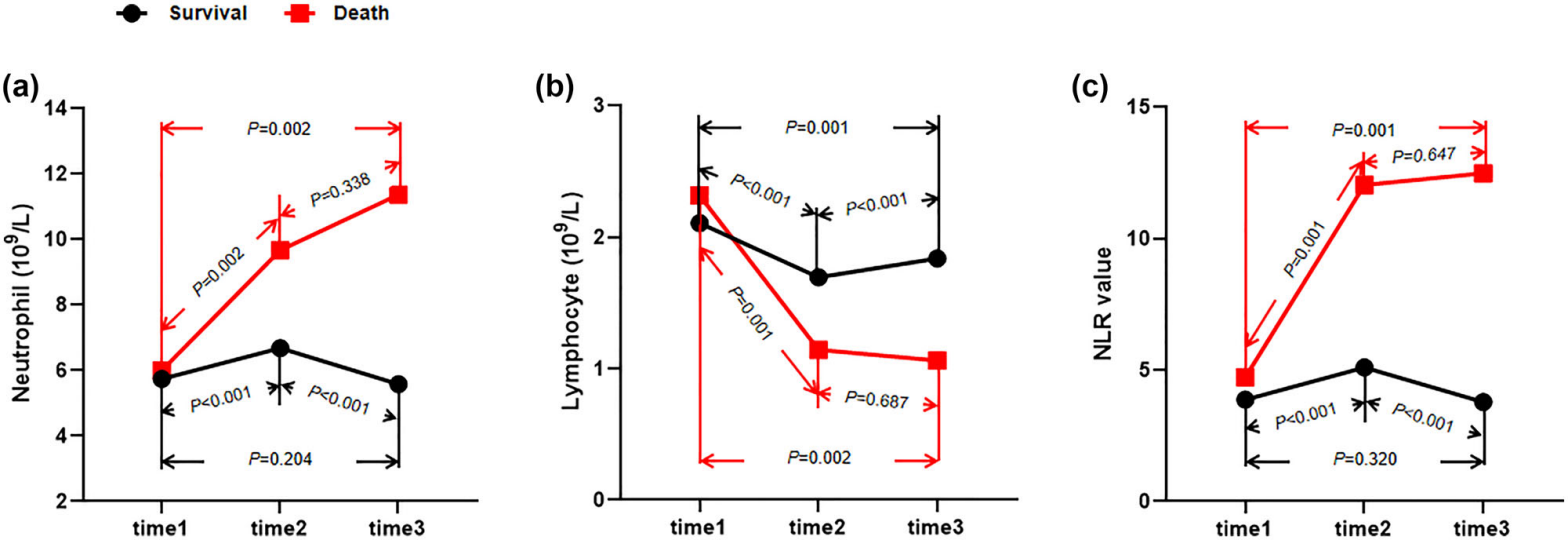
Inflammatory biomarkers at different time points between survival and death groups. (a) Showing the dynamic changes of Neutrophil levels between survival and death groups. (b) Showing the dynamic changes of lymphocyte levels between survival and death groups. (c) Showing the dynamic changes of NLR levels between survival and death groups. NLR, neutrophil‐to‐lymphocyte ratio; time1, admission; time2, 24 h after intravenous thrombolysis; time3, 12 ± 2 days after intravenous thrombolysis.

**FIGURE 5:**
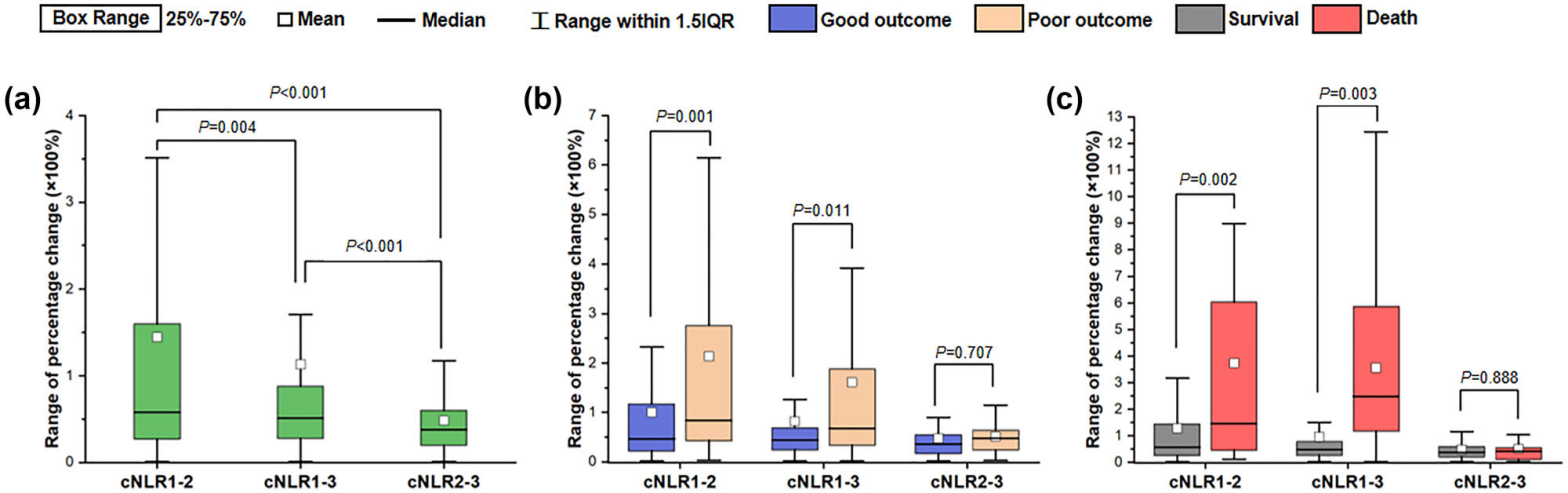
Comparison of the changes in neutrophil‐to‐lymphocyte ratio (NLR) at different time points between groups. (a) Comparison among the cNLR1‐2, cNLR1‐3 and cNLR2‐3. (b) Comparison of the changes in NLR at different time points between good and poor outcome groups. (c) Comparison of the changes in NLR at different time points between survival and death groups. cNLR1–2, percentage change of NLR between admission and 24 h after intravenous thrombolysis; cNLR1–3, percentage change of NLR between admission and 12 ± 2 days after intravenous thrombolysis; cNLR2–3, percentage change between 24 h and 12 ± 2 days after intravenous thrombolysis.

Figure 2 exhibits the changes of neutrophil, lymphocyte, and NRI after IVT in all patients. Neutrophil and NRI significantly increased at 24 h after IVT and then declined, whereas lymphocyte occurred the opposite changes. Higher neutrophil and NLR and lower lymphocyte were found at 24 h and 12 days after IVT in poor outcome versus good outcome group (Figure 3) or death versus survival group (Figure 4). Furthermore, cNLR1–2 was most obvious change among three time points, whereas cNLR1–2 and cNLR1–3 were significantly associated with poor outcome or death (Figure 5).

### 3.3 Logistic analysis of predictors associated with poor outcome or death

In univariate analysis, weight, BMI, atrial fibrillation, TOAST subtype, admission NIHSS score, NLR2, NLR3, cNLR1–2, and cNLR1–3 were found to be associated with poor outcome, whereas atrial fibrillation, admission NIHSS score, heart rate, NLR2, NLR3, cNLR1–2, and cNLR1–3 were found to be associated with death (Table 3).

**TABLE 3:** Univariate analysis of predictors associated with 3‐month poor outcome or death.

	Poor outcome			Death		
Variable	OR	95%CI	*p*	OR	95%CI	*p*
Age (years)	1.012	.991–1.033	.280	1.043	.999–1.090	.055
Gender (F/M)	.827	.487–1.404	.482	.738	.275–1.977	.545
Height (cm)	.980	.947–1.014	.242	.968	.908–1.032	.317
Weight (kg)	.969	.949–.990	.004	.980	.942–1.020	.314
BMI (kg/m^2^)	.893	.829–.962	.003	.950	.828–1.089	.462
Hypertension	.861	.519–1.428	.562	1.394	.506–3.840	.520
Diabetes mellitus	.748	.407–1.377	.351	.413	.092–1.851	.248
Coronary heart disease	1.307	.626–2.727	.476	2.089	.644–6.777	.220
Atrial fibrillation	2.082	1.054–4.113	.035	4.011	1.452–11.082	.007
Previous stroke	1.013	.562–1.826	.967	2.257	.834–6.108	.109
Current smoking	1.058	.638–1.754	.827	.533	.184–1.543	.246
Current drinking	1.005	.562–1.795	.987	2.711	.606–12.131	.192
TOAST subtype						
LAA			.001^a^			.921^a^
CE	.502	.135–1.872	.305	.811	.093–7.066	.849
SAO	.773	.196–3.054	.714	–	–	–
SOE	.164	.041–.660	.011	–	–	–
SUE	1.333	.088–20.108	.835	1.340	.147–12.260	.795
Admission NIHSS score	1.118	1.075–1.161	<.001	1.081	1.037–1.128	<.001
Heart rate (/min)	1.015	.999–1.031	.059	1.030	1.005–1.057	.020
SBP (mmHg)	1.007	.996–1.018	.244	1.007	.986–1.028	.526
DBP (mmHg)	1.001	.984–1.019	.903	.991	.957–1.026	.602
random blood glucose (mmol/L)	1.013	.958–1.070	.655	.994	.888–1.112	.913
NLR (neutrophil to lymphocyte ratio)						
NLR1 (on admission)	1.047	.984–1.113	.145	1.041	.948–1.144	.402
NLR2 (24 h after IVT)	1.276	1.166–1.397	<.001	1.193	1.104–1.290	<.001
NLR3 (12 ± 2 days after IVT)	1.306	1.170–1.457	<.001	1.288	1.168–1.420	<.001
cNLR1–2 (×100%)	1.308	1.121–1.526	.001	1.248	1.082–1.441	.002
cNLR1–3 (×100%)	1.236	1.050–1.454	.011	1.315	1.100–1.572	.003
cNLR2–3 (×100%)	1.062	.775–1.456	.707	1.039	.607–1.780	.888

Abbreviations: CE, cardioembolism; CI: confidence interval; cNLR1–2, percentage change between NLR1 and NLR2; cNLR1–3, percentage change between NLR1 and NLR3; cNLR2–3, percentage change between NLR2 and NLR3; DBP, diastolic blood pressure; IVT, intravenous thrombolysis; LAA, large artery atherosclerosis; NIHSS, National Institute of Health Stroke Scale; OR, odds ratio; SAO, small‐artery occlusion; SBP, systolic blood pressure; SOE, stroke of other determined etiology; SUE, stroke of undetermined etiology; TOAST, Trial of Org 10 172 in acute stroke treatment.

^a^ *p* for trend.

In the multivariate regression analysis, NLR2 (OR: 1.182, 95%CI: 1.180–1.293, *p* < .001) and NLR3 (OR: 1.218, 95%CI: 1.087–1.365, *p =* .001) were the independent predictors of 3‐month poor outcome, whereas NLR2 (OR: 1.17, 95%CI: 1.078–1.269, *p* < .001), NLR3 (OR: 1.252, 95%CI: 1.136–1.380, *p* < .001), cNLR1–2 (OR: 1.214, 95%CI: 1.014–1.416, *p =* .014), and cNLR1–3 (OR: 1.233, 95%CI: 1.053–1.445, *p =* .009) were independent predictors of poor outcome or death (Table 4). According to the cutoff points calculated based on the ROC curve, the multivariate analysis showed that NLR2 (OR: 3.627, 95%CI: 1.927–6.828, *p* < .001), NLR3 (OR: 4.234, 95%CI: 2.275–7.882, *p* < .001), cNLR1–2 (OR: 2.539, 95%CI: 1.413–4.562, *p =* .002), and cNLR1–3 (OR: 3.709, 95%CI: 1.739–7.907, *p =* .001) were associated with poor outcome (Table 4). The similar results were identified in death versus survival group (Table 4).

**TABLE 4:** Multivariate analysis of predictors associated with 3‐month poor outcome or death.

	Poor outcome				Death		
Variable	OR	95%CI	*p*	Variable	OR	95%CI	*p*
NLR2	1.182	1.180–1.293	**<.001**	NLR2	1.170	1.078–1.269	**<.001**
NLR3	1.218	1.087–1.365	**.001**	NLR3	1.252	1.136–1.380	**<.001**
cNLR1–2 (×100%)	1.176	.993–1.393	.060	cNLR1–2 (×100%)	1.214	1.014–1.416	**.014**
cNLR1–3 (×100%)	1.105	.948–1.288	.203	cNLR1–3 (×100%)	1.233	1.053–1.445	**.009**
^a^NLR2 (≥4.91)	3.627	1.927–6.828	**<.001**	^a^NLR2 (≥8.91)	8.806	2.931–26.463	**<.001**
^a^NLR3 (≥3.94)	4.234	2.275–7.882	**<.001**	^a^NLR3 (≥7.72)	23.948	7.024–81.653	**<.001**
^a^cNLR1–2 (≥0.68)	2.539	1.413–4.562	**.002**	^a^cNLR1–2 (≥0.73)	2.958	.954–9.173	.060
^a^cNLR1–3 (≥1.13)	3.709	1.739–7.907	**.001**	^a^cNLR1–3 (≥1.17)	20.071	5.357–75.201	**<.001**

*Note*: Poor outcome adjusted for age, BMI, atrial fibrillation, heart rate, admission NIHSS score, TOAST subtype. Dead adjusted for age, atrial fibrillation, heart rate, admission NIHSS score.

Abbreviations: CI: confidence interval; cNLR1–2, percentage change between NLR1 and NLR2; cNLR1–3, percentage change between NLR1 and NLR3; NIHSS National institutes of Health Stroke Scale; NLR, neutrophil‐to‐lymphocyte ratio; NLR1, admission NLR; NLR2, NLR at 24 h after intravenous thrombolysis; NLR3, NLR at 12 ± 2 days after intravenous thrombolysis; OR, odds ratio.

^a^ As categorical variable by dichotomizing from the cut‐off points identified in receiver operating characteristic analysis.

### 3.4 ROC curve analysis for poor outcome or death

**FIGURE 6:**
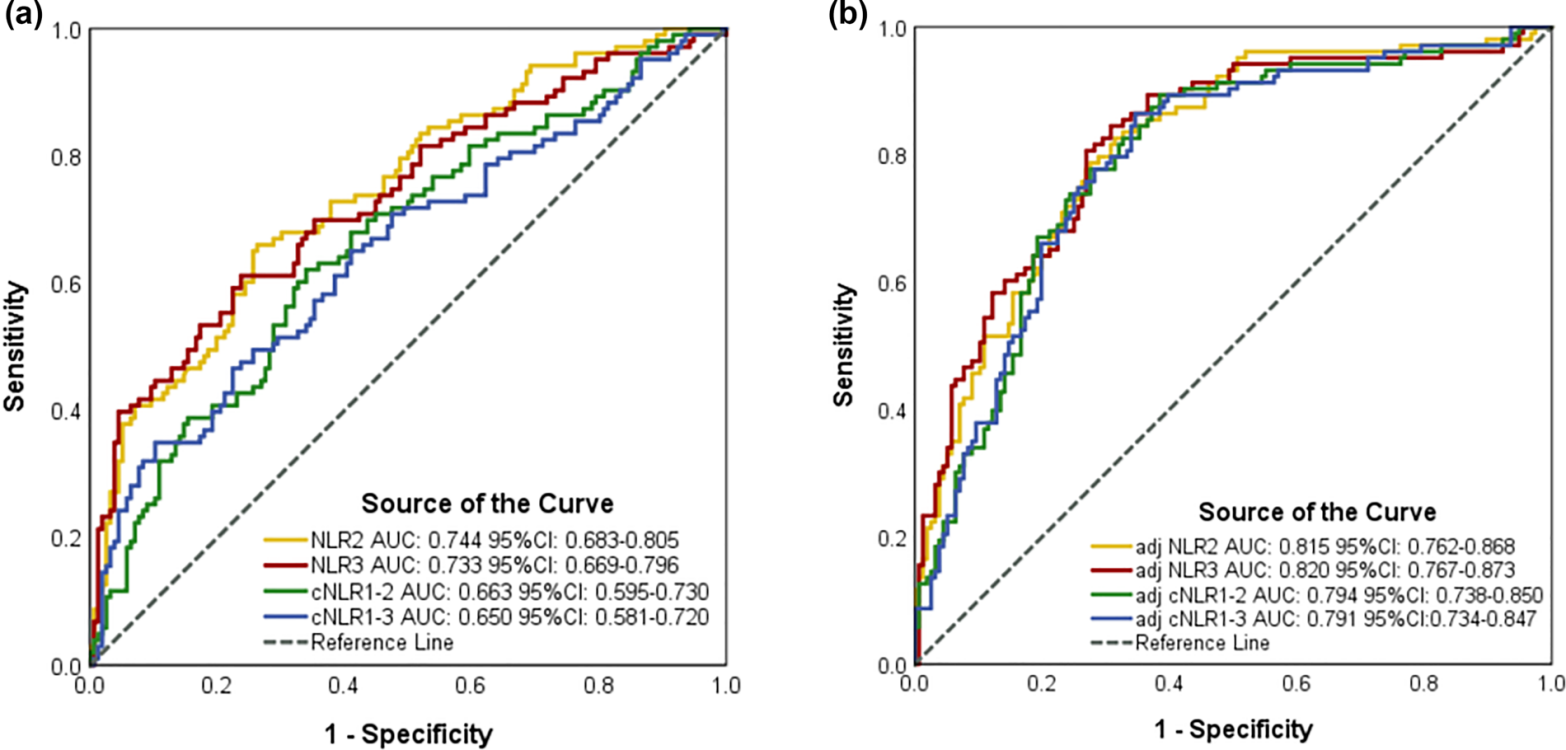
Receiver operating characteristic curve (ROC) analysis of inflammatory biomarkers to predict 3‐month poor outcome (A, unadjusted; B, adjusted). Adjusted by age, body mass index, heart rate, admission National Institutes of Health Stroke Scale (NIHSS) score, TOAST subtype. AUC, area under the curve; CI, confidence interval; NLR, neutrophil‐to‐lymphocyte ratio; NLR2, NLR of 24 h after intravenous thrombolysis; NLR3, NLR of 12 ± 2 days after intravenous thrombolysis; cNLR1–2, percentage change between NLR1 and NLR2; cNLR1–3, percentage change between NLR1 and NLR3.

**FIGURE 7:**
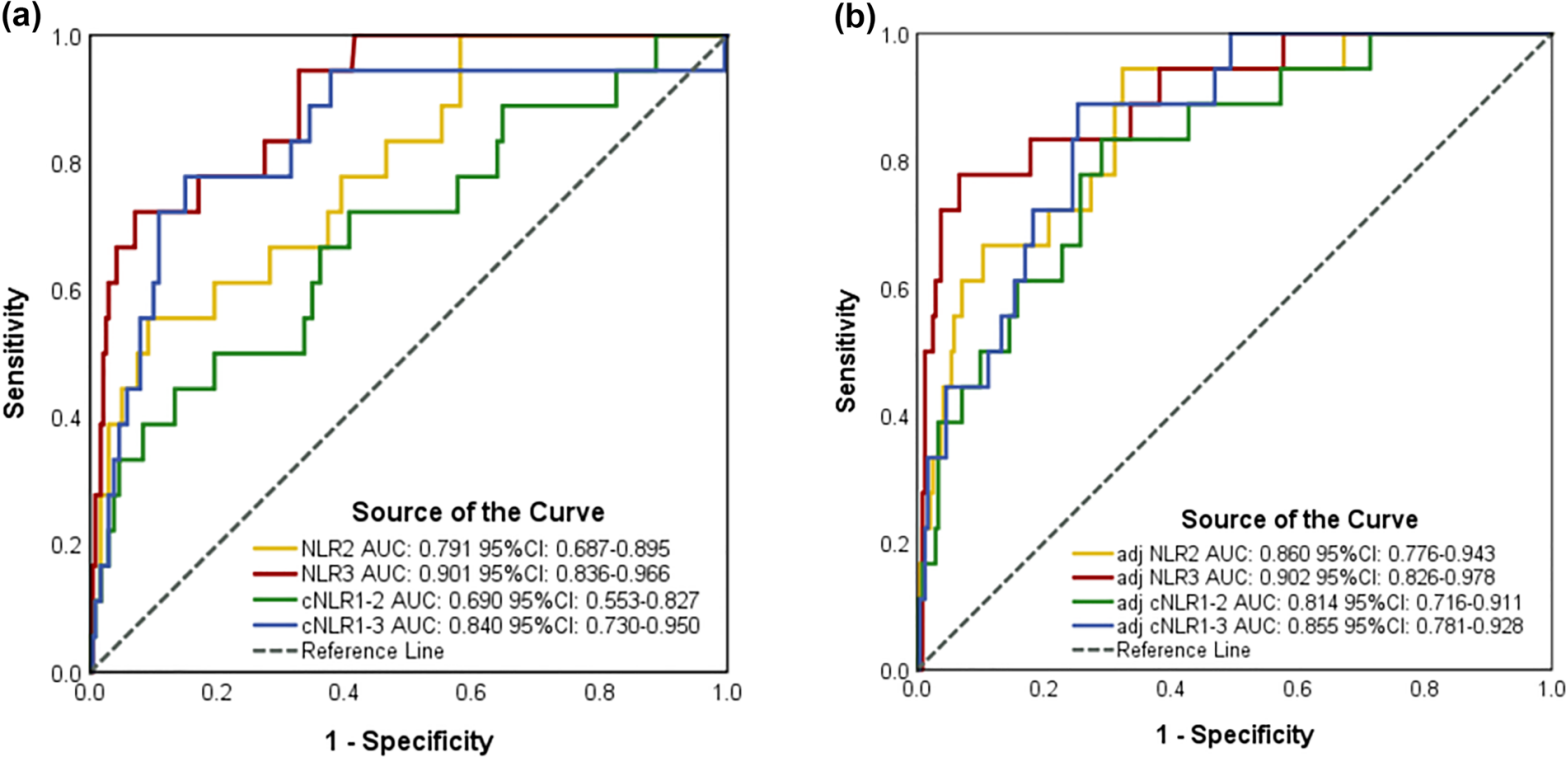
Receiver operating characteristic curve (ROC) analysis of inflammatory biomarkers to predict 3‐month death (A, unadjusted; B, adjusted). Adjusted by age, body mass index, heart rate, admission National institutes of Health Stroke Scale (NIHSS) score, TOAST subtype. AUC, area under the curve; CI, confidence interval; NLR, neutrophil‐to‐lymphocyte ratio; NLR2, NLR of 24 h after intravenous thrombolysis; NLR3, NLR of 12 ± 2 days after intravenous thrombolysis; cNLR1–2, percentage change between NLR1 and NLR2; cNLR1–3, percentage change between NLR1 and NLR3.

As to 3‐month poor outcome, ROC curve analysis showed that the AUCs for NLR2, NLR3, cNLR1–2, and cNLR1–3 were 0.815, 0.820, 0.794, and 0.791, respectively (Figure 6). As to 3‐month death, ROC curve analysis showed that the AUCs for NLR2, NLR3, cNLR1–2, and cNLR1–3 were 0.86, 0.902, 0.814, and 0.855, respectively (Figure 7).

## 4 DISCUSSION

In this study, we found that (1) there were dynamic changes of NLR in AIS after IVT; (2) NLR at 24 h and 12 days after IVT, but not at the baseline, as well as its dynamic changes were independent prognostic factors for poor outcome and death; (3) NLR and its dynamic changes have good predicting power for poor outcome and death in this population. Collectively, this is the first comprehensive study to investigate the association of NLR and its dynamic changes with outcome and death in AIS after IVT and provide evidence of their good predictive power for outcome and death.

It is already known that inflammation is implicated in the pathogenesis of stroke (Kim et al., 2016; Parikh et al., 2020; Ramiro et al., 2018). Neutrophils have been demonstrated to aggravate brain damage by releasing inflammatory mediators, aggravating oxidative stress, and increasing blood–brain barrier permeability (Stoll & Nieswandt, 2019; Zhu et al., 2018). The recruitment of neutrophils is a feature of early inflammatory response characterized with their migrating to the ischemic area (Kenne et al., 2012), and they appear within minutes of the brain injury and peak at 24–48 h, followed by a gradual reduction (Alam et al., 2020). Consistent with previous findings, our study showed that neutrophil levels were at their peak at 24 h after IVT and fell back at 12 days after IVT, and its increase was closely related to their 3‐month poor outcome and death. The lymphocyte counts as an index for general health, influenced by acute physiologic stress, were actively involved in a protective mechanism in the ischemic brain (Li et al., 2015; Liesz et al., 2013). The decrease in the lymphocyte count is related to the early neuroinflammation and the deterioration of immune function, leading to poor clinical outcomes (Yang et al., 2020). Consistent with these studies, we found that the lymphocytes had a significant decrease at 24 h after IVT, which gradually recovered at 12 days after IVT, and its decrease was associated with poor outcome and death.

NLRs, calculated from neutrophils and lymphocytes, were found to be closely related to the effects of neutrophils and lymphocytes on stroke and their interaction mechanisms (Brooks et al., 2014; Li et al., 2022; Parikh et al., 2020; Ramiro et al., 2018). Prior studies showed that NLR was used to predict the prognosis of patients with AIS after thrombolysis in stroke patients (Brooks et al., 2014; Li et al., 2022; Maestrini et al., 2015). Most studies have collected the NLR value at several time points within 7 days after IVT (generally at admission, 24, 48 h, and 7 days) (Chu et al., 2020; Guo et al., 2016) and found that NLR level increased after stroke onset and reached the peak at 12–48 h (Li et al., 2022; Sadeghi et al., 2022; Shi et al., 2018; Weng et al., 2021). In agreement with these studies, we found that NLR peaked at 24 h after IVT. Furthermore, we found that NLR returned to baseline levels around 12 days after IVT, which was reported previously. In addition, we found NLR 24 h and 12 days after IVT and its change from the baseline are closely related to the 3‐month outcome and death of AIS patients.

The strength of this study was to comprehensively explore the association of NLR dynamic change with clinical outcome in patients with AIS after IVT based on a prospective cohort. We found that NLR changes between admission and 24 h after IVT, and admission and 12 days after IVT are closely related to the 3‐month outcome and mortality of AIS patients. Furthermore, we provided the optimal cutoff of NLR at 24 h, 12 days after IVT to predict 3‐month poor outcome and mortality, which may assist in outcome stratification in this population and future clinical trial design.

### 4.1 Limitations

Our study has several limitations. First, the main limitation is the retrospective nature of analysis, which inevitably resulted in the selection bias and confounding factors. Second, only 259 patients were included from about 40 centers in the current study. The relatively small sample may introduce a potential selection bias. Third, only patients who received IVT and collected blood routine examination at three time points were included in our analysis, so we were unable to investigate the effect of NLR in different populations with IVT, endovascular or standard stroke care without reperfusion treatment, and compare the predictive value of NLR versus other inflammatory biomarkers, such as CRP, IL‐6, and TNF‐alpha, in this population. Thus, the findings warrant further confirmation in prospective trials with large sample.

## 5 CONCLUSION

This comprehensive study showed that NLR at 24 h and 12 days after IVT, and its dynamic change were independent prognostic factors for 90‐day clinical outcomes in AIS patients after IVT with good predictive powers.

## AUTHOR CONTRIBUTIONS

Hui‐Sheng Chen supervised the design. Qiong Wu conducted the analyses and drafted the manuscript. Hui‐Sheng Chen critically revised the manuscript.

## CONFLICT OF INTEREST STATEMENT

The authors declare no conflicts of interest.

### PEER REVIEW

The peer review history for this article is available at https://publons.com/publon/10.1002/brb3.3162.

## Data Availability

Data are available on reasonable request.
